# PBK drives PARP inhibitor resistance through the TRIM37/NFκB axis in ovarian cancer

**DOI:** 10.1038/s12276-022-00809-w

**Published:** 2022-07-20

**Authors:** Hanlin Ma, Gonghua Qi, Fang Han, Jiali Peng, Cunzhong Yuan, Beihua Kong

**Affiliations:** 1 Department of Obstetrics and Gynecology, Qilu Hospital of Shandong University, Jinan, 250012 China; 2 Gynecologic Oncology Key Laboratory of Shandong Province, Qilu Hospital of Shandong University, Jinan, 250012 China; 3 School of Medicine, Cheeloo College of Medicine, Shandong University, Jinan, 250012 China; 4 Department of Ophthalmology, Qilu Hospital of Shandong University, Jinan, 250012 China

**Keywords:** Ovarian cancer, Cancer therapeutic resistance, Mechanisms of disease, Translational research

## Abstract

Resistance to PARP inhibitors (PARPi) remains a therapeutic challenge in ovarian cancer patients. PDZ-binding kinase (PBK) participates in the chemoresistance of many malignancies. However, the role of PBK in PARPi resistance of ovarian cancer is obscure. In the current study, we demonstrated that overexpression of PBK contributed to olaparib resistance in ovarian cancer cells. Knockdown of PBK sensitized olaparib-resistant SKOV3 cells to olaparib. Inhibition of PBK using a specific inhibitor enhanced the therapeutic efficiency of olaparib. Mechanically, PBK directly interacted with TRIM37 to promote its phosphorylation and nuclear translocation. which subsequently activates the NFκB pathway. Additionally, PBK enhanced olaparib resistance of ovarian cancer by regulating the NFκB/TRIM37 axis in vitro and in vivo. In conclusion, PBK confers ovarian cancer resistance to PARPi through activating the TRIM37-mediated NFκB pathway, and targeted inhibition of PBK provided the new therapy to improve PARPi treatment outcomes for ovarian cancer patients.

## Cancer: Enzyme confers resistance to targeted chemotherapy

An enzyme implicated in tumor progression also helps cancers thwart a commonly used type of targeted drug therapy. Beihua Kong and colleagues from Shandong University, Jinan, China, showed how PDZ-binding kinase (PBK), an enzyme that promotes the proliferation and spread of cancer cells, activates a signaling pathway that renders tumors resistant to treatment with olaparib. This precision anti-cancer drug works by blocking a protein called PARP that normally helps cells repair damaged DNA. The researchers showed how PBK interacts with another protein to stimulate a transcription factor previously shown to reduce the effectiveness of radiation and chemotherapy. Blocking the activity of PBK, either pharmacologically or genetically, enhanced the sensitivity of ovarian cancer cells to olaparib. A similar drug strategy could help improve outcomes for cancer patients undergoing PARP inhibitor treatment.

## Introduction

Ovarian cancer ranks as the most lethal gynecologic malignancy worldwide^1^. High-grade serous ovarian carcinoma (HGSOC) accounts for the most common histologic subtype of epithelial ovarian cancer, which is characterized by 70% of patients having advanced disease at diagnosis and poor prognosis. Traditional platinum and paclitaxel-based chemotherapy have limited efficacy with 70% of patients relapsing within two years^2^. Recently, PARP inhibitors (PARPi) represented by olaparib have been approved for the maintenance therapy of advanced ovarian cancer after response to first-line chemotherapy^3^. Although PARPi maintenance therapy significantly extends the progression-free survival (PFS) in ovarian cancer patients, an increasing number of women develop primary or acquired resistance to PARPi^4^. Thus, it possesses significant clinical value to investigate the mechanisms of resistance to PARPi and construct strategies for subsequent combination therapies.

PDZ-binding kinase (PBK), also known as T-lymphokine-activated killer-cell-originated protein kinase (TOPK), is a serine/threonine kinase that participates in cell cycle regulation and mitotic progression^5,6^. Notably, PBK is an oncogenic protein that plays vital roles in the proliferation, progression, and metastasis of many malignancies^7–11^. The expression of PBK is abundant in various cancers compared with in normal tissues, making it a promising target in diagnosis and prognosis prediction, as well as targeted therapy of malignancy^12^. Moreover, PBK is confirmed to confer resistance against chemotherapy and radiotherapy in various types of cancers. PBK hampers paclitaxel-induced autophagic cell death through negative regulation of p53 activity and expression in non-small cell lung cancer cells (NSCLC)^13^. The COX2-TXA_2_ pathway promotes MET transcription through MET-mediated PBK phosphorylation at the Y74 site, resulting in gefitinib resistance in NSCLC cells^14^. Overexpression of PBK impairs oxaliplatin-induced apoptosis by the PTEN/AKT axis in hepatocellular carcinoma cells^15^. PBK contributes to tumor cell development and doxorubicin resistance via physically interacting with p53 and downregulating its function^16^.

PBK has also been shown to be overexpressed in cervical cancer and confer resistance to cisplatin treatment through regulating ERK/c-Myc signaling pathway^17^. Our previous study demonstrates that PBK is highly expressed and correlates with platinum resistance in patients with HGSOC. The up-regulated PBK contributes to cisplatin resistance by promoting cellular autophagy in ovarian cancer^18^. However, the role of PBK in the PARPi resistance of ovarian cancer remains unclear. In the current study, we test whether PBK is involved in the regulation of PARPi resistance and try to clarify the underlying mechanisms.

## Materials and methods

### Cell lines and cell culture

The UWB1.289 and SKOV3 cell lines were obtained from the American Type Culture Collection (ATCC, Manassas, VA, USA). HEK293T cell line was obtained from the Cell Bank of the Chinese Academy of Sciences (Shanghai, China). Olaparib-resistant SKOV3 (SKOV3/Ola) cells were generated by exposing SKOV3 cells to increasing concentrations of olaparib in our lab. All cell lines were authenticated by short tandem repeat (STR) profiling. UWB1.289 cells were maintained in RPMI 1640 medium (Gibco, Grand Island, NY, USA). HEK293T was cultured in Dulbecco’s modified Eagle’s medium (DMEM). SKOV3 and SKOV3/Ola cell lines were maintained in McCoy’s 5A medium. All media had 10% fetal bovine serum (FBS, Gibco) and 1% penicillin/streptomycin (15140-122, Gibco). All cells were cultured in a humidified incubator at 37 °C with 5% CO_2_.

### Antibodies and reagents

Antibodies against GST (10000-0-AP), poly (ADP-ribose) polymerase 1 (PARP1, 13371-1-AP), Flag (20543-1-AP), MYC-tag (16286-1-AP), GFP (66002-1-Ig), Ki-67 (27309-1-AP), PBK (16110-1-AP), XIAP (10037-1-Ig), Bcl-XL (26967-1-AP), Lamin B1 (12987-1-AP), p65 (66535-1-Ig), and caspase 3 (19677-1-AP) were purchased from Proteintech (Wuhan, China); β-actin (A5441) was obtained from Sigma-Aldrich (St. Louis, MO, USA); The mouse IgG (A7028) and rabbit IgG (A7016) were purchased from Beyotime (Shanghai, China). Antibodies for IκBα (ab32518), TRIM37 (ab264190), and GAPDH (ab8245) were obtained from Abcam (Cambridge, UK). Cleaved caspase 3 (9661) and p-IκBα (2859) were obtained from Cell Signaling Technology (Danvers, MA, USA). p-Thr (sc-5267), p-Ser (sc-81514), and p-Tyr (sc-7020) were purchased from Santa Cruz (Dallas, TX, USA). Goat anti-mouse IgG Alexa Fluor-594 (A-11030) for immunofluorescence staining was obtained from Invitrogen (Waltham, MA, USA). Olaparib (AZD2281), JSH-23 (S7652), and OTS514 (S7652) were purchased from Selleck Chemicals (Houston, TX, USA).

### Western blot

Western blot assay was conducted as previously described^18^. Briefly, cells were rinsed with PBS and resuspended in Western and IP Lysis Buffer (P0013, Beyotime) with 1 mM PMSF. The cell supernatants were collected after centrifugation at 12,000 rpm for 5 min. Protein samples were quantified using the BCA Protein Assay Kit (P0012, Beyotime). A total of 40–80 µg proteins were separated using 8–15% gradient SDS-PAGE gels and transferred onto PVDF membranes (Merck Millipore, Burlington, MA, USA). The membranes were then incubated with 5% fat-free milk, primary antibodies (1:1000), and appropriate HRP-conjugated secondary antibodies (1:5000). Protein expression was visualized using an enhanced ECL detection kit (ORT2655, PerkinElmer, Waltham, MA, USA) and GE Amersham Imager 600 (GE, Chicago, IL, USA). The gray values of the bands were analyzed using ImageJ 1.52a.

### Co-immunoprecipitation (Co-IP)

Cells were washed twice with PBS and lysed using Western and IP Lysis Buffer, then centrifuged at 12000 rpm for 5 min. Next, 800 μg of the cellular extract was incubated with primary antibodies (10 µL) or normal rabbit/mouse IgG overnight, then mixed with protein A/G agarose beads (P2012, Beyotime) for 2 h in a rotary shaker. After washing the beads three times with PBS containing 1 mM PMSF, the captured immune complexes were resuspended with 2 × SDS loading buffer and subjected to western blot assay.

### Plasmid construction and transfection

The PCMV-PBK and PCMV-TRIM37 plasmids were generated by inserting the open reading frames (ORFs) of human PBK and TRIM37 into the PCMV vector (PS100069, OriGene, Rockville, MD, USA). The pMyc-C2-PBK, pMyc-C2-TRIM37, pEGFP-C2-PBK, and pEGFP-C2-TRIM37 plasmids were generated by cloning the ORFs of PBK and TRIM37 into the pMyc-C2 and pEGFP-C2 vectors, respectively. PBK shRNA-1 (TRCN0000001806), PBK shRNA-2 (TRCN0000001805), TRIM37 shRNA-1 (TRCN0000349177), and TRIM37 shRNA-2 (TRCN0000040783) vectors were purchased from Sigma-Aldrich. All constructs were validated using DNA sequencing. Plasmid transient transfection was performed using a Lipofectamine 2000 reagent (11668-019, Invitrogen) according to the manufacturer’s instructions. Lentivirus particles were produced in HEK293T cells packaged with psPAX2 and pMD2.G. To obtain stable transfection cell lines, cells were infected with lentivirus for 24 h and then selected for two weeks in corresponding media containing 2 μg/mL puromycin (P8833, Sigma-Aldrich).

### Annexin V/PI assay for apoptosis

Treated cells (1 × 10^6^) were washed with PBS, and stained with FITC-Annexin V (5 µL) and propidium iodide (PI, 5 µL) in 1×Binding Buffer (556547, BD Bioscience, Franklin Lakes, NJ, USA) for 15 min in the dark. The stained cells were acquired using a BD FACSCalibur flow cytometer instrument and analyzed using FlowJo v10.6.2 software.

### Colony formation assay

Cells (600 cells/well) were plated in six-well plates as single-cell suspensions. After olaparib treatment for 7–14 days, the colonies were fixed with methanol for 15 min and stained with crystal violet for 15 min. The number of colonies (>50 cells) was counted using ImageJ 1.52a software.

### MTT assay

Cells were seeded onto 96-well plates at a concentration of 3000 cells per well. After treatment, a 20 µL MTT solution was added into each well. Four hours later, the medium was removed, and 200 µL DMSO was added. The plate was then gently vortexed in the dark for 10 min. The optical density (OD) value was determined by measuring the absorbance at 490 nm using a Thermo Scientific Microplate Reader (Waltham, MA, USA).

### Immunohistochemistry (IHC) staining

Tumor tissues were formalin fixed, paraffin embedded, cut into 4 μm tissue slices, and then processed using standard deparaffinization and rehydration techniques. After antigen retrieval using EDTA solution in a microwave, the slices were incubated with primary antibodies (1:100) overnight and stained using a Universal IHC SP kit (SP9000, ZSGB-BIO, Beijing, China). Next, the slices were visualized using DAB (ZSGB-BIO) and counterstained with hematoxylin. Slices were imaged using an Olympus microscope (Tokyo, Japan).

### Immunofluorescence staining

Cells were seeded into 24-well plates that were pre-inserted with glass slides. After receiving indicated treatment, the cells were fixed with 4% paraformaldehyde for 15 min, followed by permeabilization with 0.2% Triton X-100 solution for 5 min. Next, the cells were blocked with normal goat serum for 30 min, incubated with p65 primary antibodies (1:100) overnight, and Goat anti-mouse IgG Alexa Fluor-594 secondary antibody (1:200) for 1 h. DAPI was used to indicate the nucleus. The slides were covered with antifade mounting medium and coverslips. Images were captured with an X81 microscope (Olympus).

### Mass spectrometry (MS) assay

UWB1.289 (1 × 10^7^) cells treated with or without olaparib were lysed in Western and IP Lysis Buffer. After centrifugation for 15 min, the cell supernatant was immunoprecipitated (IPed) with anti-rabbit IgG or anti-PBK antibody. Fractions of the IPed proteins were collected and separated on SDS-PAGE gels, coomassie brilliant blue stained, and subjected to liquid chromatography-tandem mass spectrometry (LC-MS/MS) sequencing and data analysis by Applied Protein Technology (Shanghai, China).

### qRT-PCR

Total RNA samples were prepared using TRIzol reagent (15596018, Invitrogen). The mRNA was subjected to reverse transcription by the PrimeScript RT reagent Kit (RR037A, TaKaRa, Kyoto, Japan). Real-time PCR was performed using 10 μL SYBR Premix Ex Taq (RR420A, TakaRa) system with the 7900HT Fast Real-Time PCR System (Applied Biosystems, Waltham, MA, USA). The mRNA levels of specific genes were normalized against β-actin using the comparative Ct method (2^−ΔΔCt^). The primer sequences for PBK: forward, 5′-GAAGAGGACTGAGAGTGGCT-3′, reverse, 5′-CTTCTGCATAAACGGAGAGGC-3′; the primer sequences for β-actin: forward, 5′-CATGTACGTTGCTATCCAGGC-3′, reverse, 5′- CTCCTTAATGTCACGCACGAT-3′.

### GST pull-down assay

The PBK ORF was inserted into the pGEX-4T-1 vector to create the GST-PBK vector. GST fusion constructs were amplified in *Escherichia coli* BL21 cells, and fusion protein lysates were prepared using BeyoGold™ GST-tag Purification Resin (P2250, Beyotime). UWB1.289 cells (1 × 10^7^) expressing PCMV-TRIM37 (flag-tag) were lysed using Western and IP Lysis Buffer and the cell supernatant was collected after centrifugation. In GST pull-down assays, 25 μg of the appropriate GST fusion proteins were mixed with 20 μL of cell supernatant and incubated in GST pull-down protein binding buffer containing GST-tag Purification Resin for 2 h. GST-tag Purification Resins were then washed twice with washing buffer, boiled in 30 μL of 2 × SDS-PAGE loading buffer, and resolved on 12% gel.

### Nuclear and cytoplasmic protein extraction

Nuclear and cytoplasmic protein separation was performed using NE-PER Nuclear and Cytoplasmic Extraction Reagents (78833, Thermo Scientific) according to the manufacturer’s instructions.

### In vivo tumor xenograft animal model

All experimental protocols were conducted within Shandong University guidelines for animal research and were approved by the Institutional Animal Care and Use Committee. UWB1.289 cells stably infected with PCMV (Ctr) and PCMV-PBK lentivirus were washed with PBS and resuspended in PBS. Cells (5 × 10^6^) were subcutaneously injected into the flanks of BALB/c nude mice (female, 4–6 weeks) which were purchased from the Gempharmatech Co., Ltd (Nanjing, China). When the tumor volumes reached nearly 50 mm^3^, mice were randomly divided into three groups (PCMV + olaparib, PCMV-PBK + olaparib, and PCMV-PBK + olaparib + JSH-23; six mice per group). Tumor bearing mice were then treated with an intraperitoneal injection of olaparib (50 mg/kg) or/and JSH-23 (5 mg/kg) every day, respectively. Fifteen days post injection, the mice were euthanized and the xenograft tumor was removed for further analysis. The tumor volumes were qualified as length × width^2^ × 0.5.

### Statistical analysis

All experiments were independently performed in triplicate unless otherwise noted. Results were reported as the means ± the standard error of the mean (SEM). Statistical significance was assessed using the Student’s *t*-test for comparison between the two groups, and one-way ANOVA tests for three or more groups (GraphPad Software, La Jolla, CA, USA). *P* < 0.05 was defined as statistically significant.

## Results

### PBK drives olaparib resistance in ovarian cancer cells

**Fig. 1:**
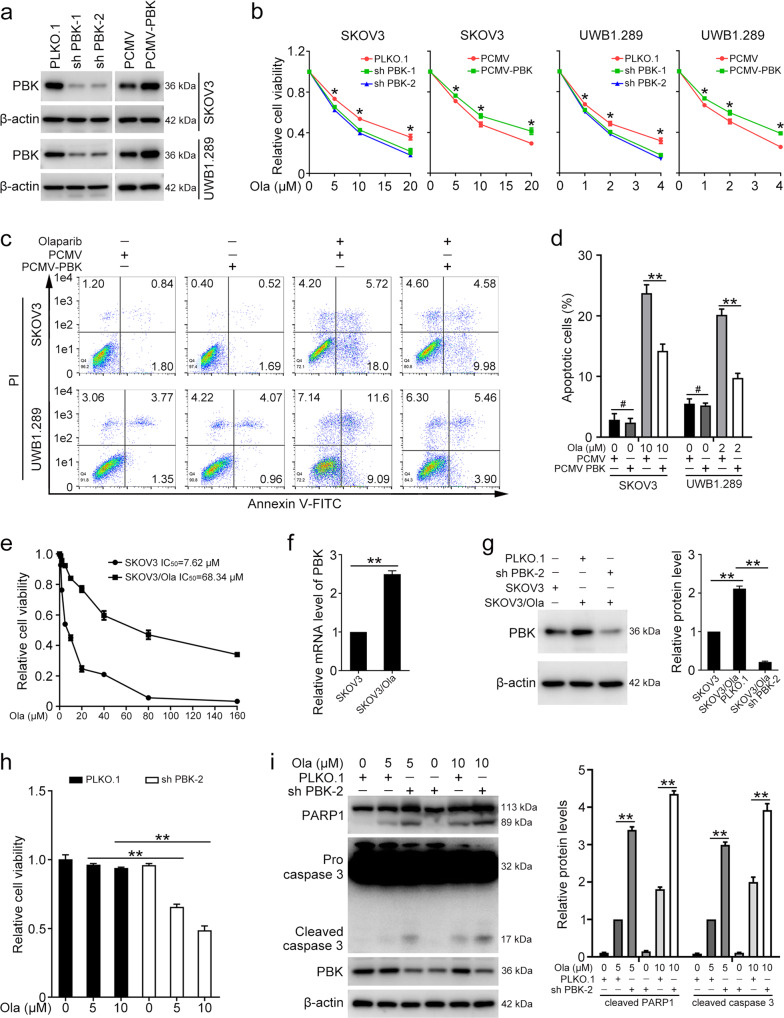
PBK promotes olaparib resistance in ovarian cancer cells. PLKO.1, PBK shRNA 1 (sh PBK-1), PBK shRNA 2 (sh PBK-2), PCMV, and PCMV-PBK plasmids were stably transfected into SKOV3 and UWB1.289 cells. **a** Western blot was performed to determine PBK protein levels. **b** The MTT assay was used to detect cell viability in cells treated with olaparib (Ola) for 72 h. **c** Flow cytometry assay was performed to detect cell apoptosis in cells treated with olaparib (SKOV3, 10 µM; UWB1.289, 2 µM) for 72 h. **d** Quantification of the proportion of apoptotic cells in **c**. **e** SKOV3 and olaparib-resistant SKOV3 (SKOV3/Ola) cells were treated with different concentrations of olaparib (Ola; 0, 1, 2, 5, 10, 20, 40, 80, 160 μM) for 72 h. The MTT assay was performed to detect cell viability. **f** qRT-PCR was used to determine the mRNA levels of PBK in SKOV3 and SKOV3/Ola cells. **g** SKOV3/Ola cells were stably transfected with PLKO.1 or PBK shRNA 2 (sh PBK-2). The protein levels of PBK and β-actin in SKOV3 and SKOV3/Ola cells were detected by western blot. **h** SKOV3/Ola cells were treated with 0, 5, or 10 μM olaparib for 72 h. The MTT assay was used to determine cell viability. **i** SKOV3/Ola cells with or without PBK knockdown were treated with 0, 5, or 10 μM olaparib for 72 h. Western blot was used to detect the protein levels of PARP1, caspase-3, PBK, and β-actin. (Data are presented as the mean ± SEM, ^#^*p* > 0.05, *p < 0.05, **p < 0.01, *n* = 3).

To determine whether PBK expression was involved in the sensitivity of ovarian cancer cells to PARPi, SKOV3, and UWB1.289 cells with PBK knockdown or overexpression were constructed and challenged with gradient concentrations of olaparib for 72 h (Fig. 1a). The MTT assay demonstrated that knockdown of PBK sensitizes SKOV3 and UWB1.289 cells to olaparib treatment. Consistently, overexpression of PBK conferred ovarian cancer cells resistance to olaparib (Fig. 1b). The colony formation assay drew the same conclusion that PBK promotes olaparib resistance in ovarian cancer cells (Fig. S1a, b). The flow cytometry assay showed that PBK overexpression markedly decreases the proportion of apoptotic cells following olaparib treatment (Fig. 1c, d). Additionally, the overexpression of PBK significantly reduced the protein levels of cleaved caspase 3 and cleaved PARP1 following olaparib treatment, whereas knockdown of PBK resulted in increased levels of cleaved caspase 3 and cleaved PARP1 (Fig. S1c–e). Thus, these results suggested that PBK mediates the sensitivity of ovarian cancer cells to PARPi.

### Knockdown of PBK sensitizes olaparib-resistant cells to olaparib

We further investigated the influence of PBK on olaparib sensitivity in olaparib-resistant SKOV3 cells (SKOV3/Ola). SKOV3/Ola and parental SKOV3 cells were exposed to different concentrations of olaparib for 72 h and the IC_50_ values were calculated. The IC_50_ value of parental SKOV3 cells was 7.62 µM, compared with 68.34 µM in SKOV3/Ola cells (Fig. 1e). The mRNA and protein levels of PBK in SKOV3/Ola cells were obviously higher than those in parental SKOV3 cells (Fig. 1f, g). SKOV3/Ola cells had almost no response to olaparib treatment (5 µM and 10 µM), whereas knockdown of PBK could make the cells sensitive to olaparib again (Fig. 1g, h). Inhibition of PBK also significantly impaired the colony formation ability of SKOV3/Ola cells upon olaparib treatment (Fig. S1f). In addition, the expression of apoptotic proteins induced by olaparib was increased in SKOV3/Ola cells with PBK knockdown compared to those in the control group (Fig. 1i). Therefore, these data indicated that PBK contributes to olaparib resistance in ovarian cancer cells.

### PBK inhibitor increases the sensitivity of ovarian cancer cells to olaparib

**Fig. 2:**
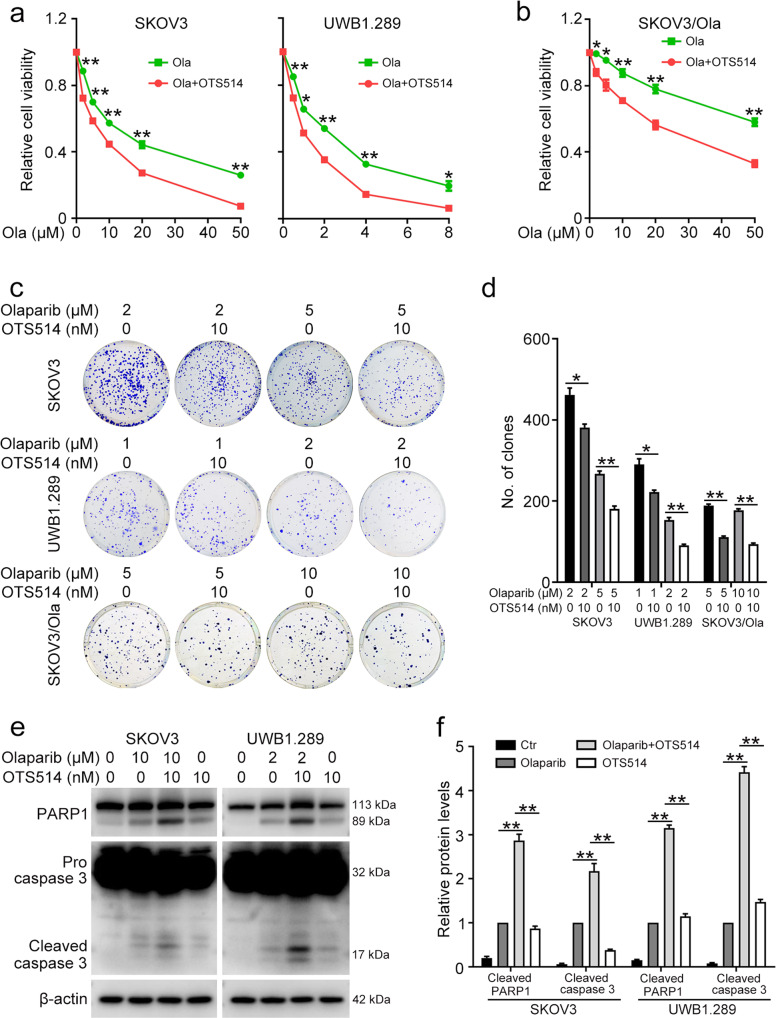
PBK inhibitor enhances the effect of olaparib. **a** The MTT assay was performed to determine the cell viability in SKOV3 and UWB1.289 cells treated with 10 nM OTS514 and olaparib for 72 h. **b** The MTT assay was performed to determine the cell viability in SKOV3/Ola cells treated with 10 nM OTS514 and olaparib for 72 h. **c** Clonogenic assay was used to assess the colony formation efficiency in cells treated with OTS514 and/or olaparib. **d** Quantification of the number of clones in **c**. **e** Western blot was used to detect the protein levels of PARP1, caspase-3, and β-actin in cells treated with OTS514 and/or olaparib. **f** Quantification of the protein levels in **e**. (Data are presented as the mean ± SEM, *p < 0.05, **p < 0.01, *n* = 3).

In order to determine the combined effect of PBK inhibitor and olaparib, a range of concentrations of olaparib was applied to SKOV3 and UWB1.289 cells in the presence of OTS514, a specific PBK inhibitor. The MTT assay showed that the application of OTS514 markedly enhances the anti-tumor effect of olaparib (Fig. 2a). Inhibition of PBK using OTS514 also significantly sensitized SKOV3/Ola cells to olaparib treatment (Fig. 2b). The combination of olaparib and OTS514 obviously decreased the colony formation abilities of SKOV3, UWB1.289, and SKOV3/Ola cells compared with the olaparib alone group (Fig. 2c, d). Moreover, OTS514 significantly aggravated the degree of apoptosis induced by olaparib treatment (Fig. 2e, f). Altogether, using the PBK inhibitor in combination with olaparib possessed the potential to overcome PARPi resistance in ovarian cancer.

### PBK promotes the phosphorylation and nuclear translocation of TRIM37

**Fig. 3:**
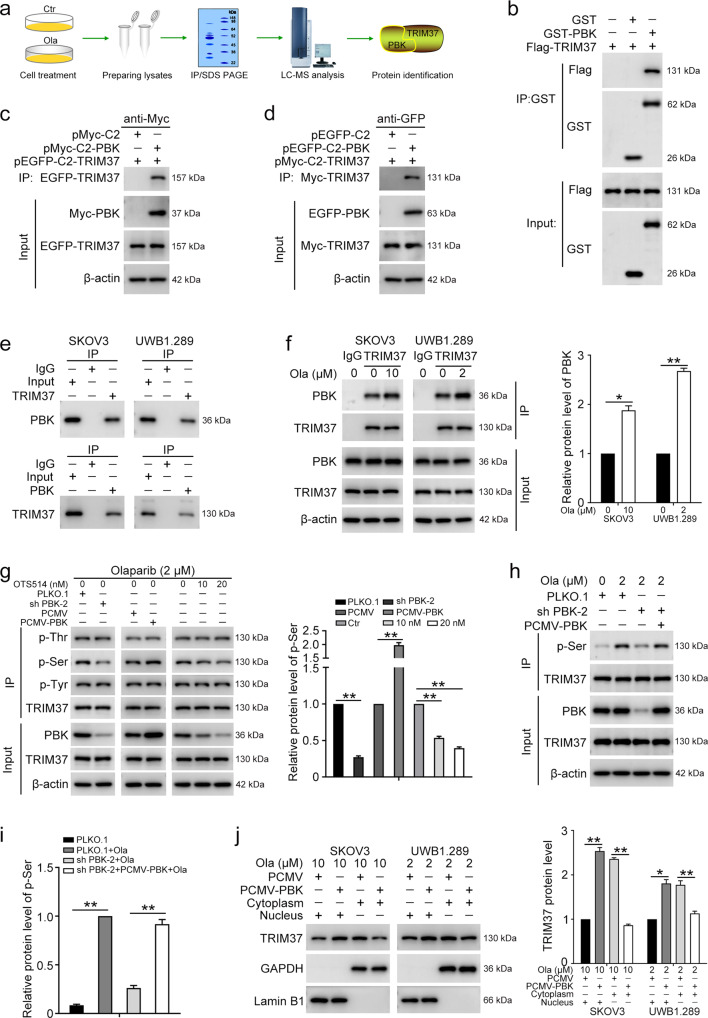
PBK directly interacts with TRIM37. **a** Experimental design of MS-based proteomic analysis. UWB1.289 (1 × 10^7^) cells were treated with or without Olaparib (Ola, 2 µM) for 24 h. Then, the cell lysates were centrifuged and the supernatant was collected. After immunoprecipitation with anti-PBK antibody, the IPed proteins were separated on SDS-PAGE gels, coomassie brilliant blue stained, and subjected to LC-MS/MS sequencing. **b** GST pull-down assay was used to validate the direct interaction between PBK and TRIM37. **c** pMyc-C2-PBK and pEGFP-C2-TRIM37 vectors were co-transfected into HEK293T cells for 48 h. Western blot was used to detect the exogenous EGFP-TRIM37 co-immunoprecipitated (Co-IPed) with anti-Myc antibody. **D** pMyc-C2-TRIM37 and pEGFP-C2-PBK vectors were co-transfected into HEK293T cells for 48 h. Western blot was used to detect the Myc-TRIM37 Co-IPed with anti-GFP antibody. **e** Co-IP was performed to verify the endogenous interaction between PBK and TRIM37 in SKOV3 and UWB1.289 cells. **f** SKOV3 and UWB1.289 cells were treated with olaparib (Ola) for 24 h, followed by pulldown with anti-TRIM37 antibody and immunoblotting with the antibodies indicated. **g** UWB1.289 cells stably transfected with PLKO.1, PBK shRNA-2 (sh PBK-2), PCMV, and PCMV-PBK were treated with OTS514 and/or olaparib (2 µM) for 24 h, followed by pulldown with anti-TRIM37 antibody and immunoblotting with p-tyrosine (p-Thr), p-serine (p-Ser), p-threonine (p-Tyr), and TRIM37 antibodies. **h** UWB1.289 cells transfected with PLKO.1, PBK shRNA 2 (sh PBK-2), and/or PCMV-PBK were stimulated with or without olaparib for 24 h, followed by pulldown with anti-TRIM37 antibody and immunoblotting with the antibodies indicated. **i** Quantification of the p-Ser level of TRIM37 in **h**. **J** Cells transfected with PCMV or PCMV-PBK were treated with olaparib for 24 h. Western blot was performed to detect the protein levels of TRIM37 in the cytoplasm and nucleus fractions. (Data are presented as the mean ± SEM, *p < 0.05, **p < 0.01, *n* = 3).

To further clarify the molecular mechanism underlying PBK-mediated olaparib resistance, we performed MS-based proteomic analysis and tripartite motif containing 37 (TRIM37) was determined to be a novel interaction partner of PBK (Fig. 3a). GST pull-down assay was introduced to confirm the direct binding between PBK and TRIM37 (Fig. 3b). Next, endogenous and exogenous Co-IP were used to further verify that TRIM37 interacts with PBK at the cellular level. The Co-IP assay demonstrated that TRIM37 binds with PBK exogenously in HEK293T cells and endogenously in SKOV3 and UWB1.289 cells (Fig. 3c–e). To verify whether the interaction between PBK and TRIM37 was related to olaparib, we treated SKOV3 and UWB1.289 cells with olaparib for 24 h. Our results indicated that olaparib treatment increases the binding between PBK and TRIM37 (Fig. 3f).

Given that the phosphorylation level of TRIM37 is crucial to its activation and nuclear translocation^19^, we then turned to explore the effect of PBK on the phosphorylation level of TRIM37. UWB1.289 cells with PBK overexpression or knockdown were exposed to olaparib and/or OTS514 for 24 h. The serine phosphorylation (p-Ser) level of TRIM37 was decreased in cells with PBK knockdown and increased in cells with PBK overexpression. Meanwhile, the PBK inhibitor also inhibited the p-Ser level of TRIM37, with no effect on tyrosine (p-Tyr) and threonine (p-Thr) phosphorylation levels (Fig. 3g). Moreover, we observed that olaparib treatment dramatically increased the p-Ser level of TRIM37. Reconstruction of PBK could rescue the decreased p-Ser level of TRIM37 that was induced by PBK knockdown (Fig. 3h, i). Furthermore, overexpression of PBK promoted the nuclear translocation of TRIM37 in SKOV3 and UWB1.289 cells (Fig. 3j). Therefore, these results indicated that PBK interacts with TRIM37 to promote its phosphorylation and nuclear translocation.

### PBK activates the NFκB pathway by interacting with TRIM37

**Fig. 4:**
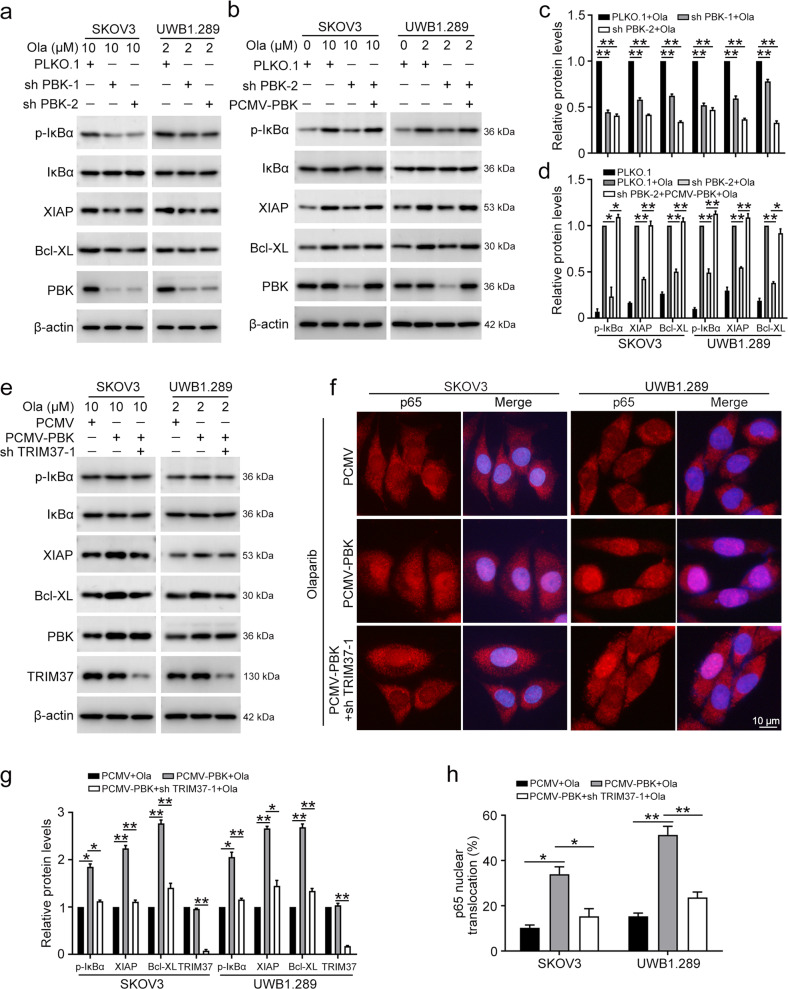
PBK activates the NFκB pathway through interacting with TRIM37. **a** SKOV3 and UWB1.289 cells transfected with PLKO.1, PBK shRNA 1 (sh PBK-1), and PBK shRNA 2 (sh PBK-2) were challenged with olaparib for 24 h, the protein levels of p-IκBα, IκBα, XIAP, Bcl-XL, PBK, and β-actin were detected using western blot. **B** Cells transfected with PLKO.1, PBK shRNA 2 (sh PBK-2), and/or PCMV-PBK were challenged with olaparib for 24 h, the protein levels were detected using western blot. **c**, **d** Quantification of the protein levels in **a** and **b**, respectively. **e** Cells transfected with PLKO.1, PBK shRNA 2 (sh PBK-2), and/or TRIM37 shRNA 1 (sh TRIM37-1) were challenged with olaparib for 24 h, the protein levels were detected using western blot. **F** After the cells were treated in the same way as **e**, immunofluorescence staining was performed to detect p65 nuclear translocation. **g** Quantification of the protein levels in **e**. **h** Quantification of the ratio of p65 nuclear translocation in **f**. (Data are presented as the mean ± SEM, *p < 0.05, **p < 0.01, *n* = 3).

Previous studies reported that TRIM37 mediates genotoxic stress-induced NFκB activation^20^, we wondered whether PBK is associated with NFκB activity through cooperating with TRIM37. We found that suppression of PBK inhibites the expression of XIAP and Bcl-XL, downstream target genes of the NFκB pathway, which play key roles in the anti-apoptosis process. The phosphorylation level of IκBα (p-IκBα) also decreased in cells with PBK knockdown, indicating the inhibition of NFκB activity (Fig. 4a, c). Meanwhile, OTS514 dramatically inhibited the expression of p-IκBα, XIAP, and Bcl-XL in ovarian cancer cells (Fig. S2). Our results also showed that olaparib treatment enhances the activation of the NFκB signaling pathway in ovarian cancer cells. Restoring the expression of PBK could reverse the decreased NFκB activity that was caused by PBK knockdown (Fig. 4b, d). In addition, overexpression of PBK conferred activation of the NFκB pathway, and this process was impeded when TRIM37 was knocked down in SKOV3 and UWB1.289 cells (Fig. 4e–h). Moreover, the TRIM37/NF-kB axis was also activated in SKOV3/Ola cells compared to the parental SKOV3 cells (Fig. S3). Therefore, PBK cooperated with TRIM37 to activate the NFκB signaling pathway in ovarian cancer.

### PBK facilitates olaparib resistance via TRIM37/NFκB pathway

**Fig. 5:**
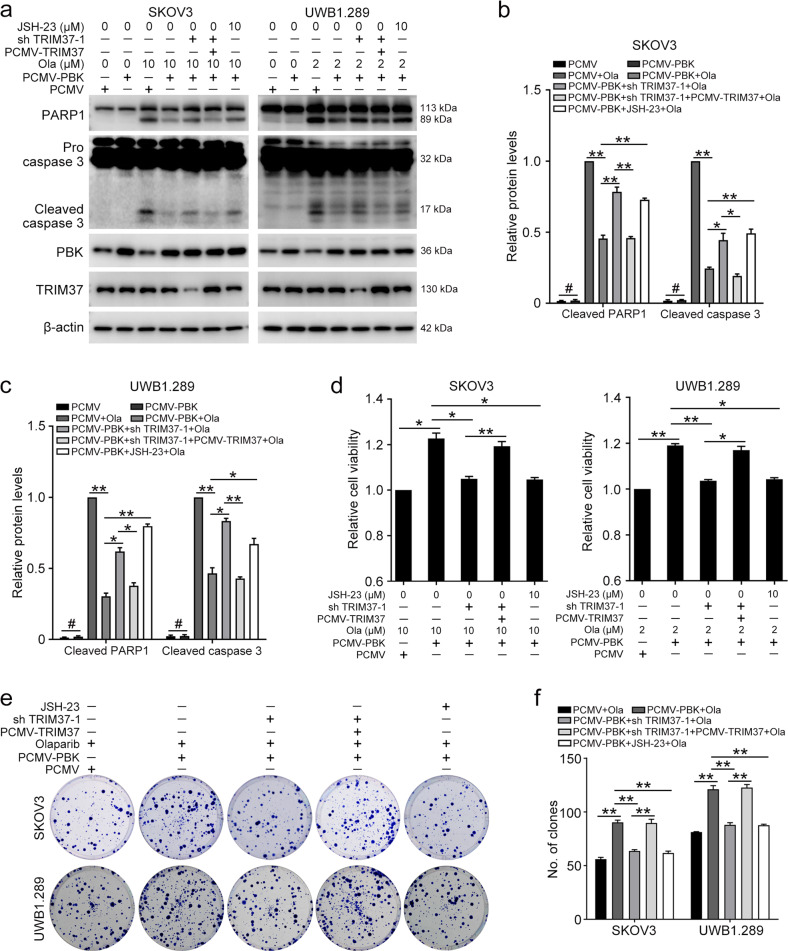
PBK promotes olaparib resistance via the TRIM37/NFκB axis. SKOV3 and UWB1.289 cells transfected with PCMV, PCMV-PBK, PCMV-TRIM37, and TRIM37 shRNA 1 (sh TRIM37-1) were challenged with olaparib for 72 h. Cells were treated with olaparib and JSH-23 together for 24 h, and then with olaparib alone for 48 h. **a** Western blot was used to detect the protein levels of PARP1, caspase-3, PBK, TRIM37, and β-actin. **b** and **c** Quantification of the protein levels in **a**. **d** The MTT assay was used to determine cell viability. **e** Clonogenic assay was used to assess the colony formation efficiency of SKOV3 and UWB1.289 cells. **f** Quantification of the number of clones in **e**. (Data are presented as the mean ± SEM, ^#^*p* > 0.05, *p < 0.05, **p < 0.01, *n* = 3).

Firstly, we explored the function of TRIM37 in the olaparib resistance of ovarian cancer cells. The MTT assay and colony formation assay both demonstrated that overexpression of TRIM37 decreases the sensitivity of ovarian cancer cells to olaparib, whereas knockdown of TRIM37 exerts the opposite effect (Fig. S4). We also pondered whether TRIM37/NFκB pathway is the key to PBK-induced olaparib resistance in ovarian cancer. Consistent with the previous research, overexpression of PBK decreased the levels of apoptotic proteins induced by olaparib treatment compared with the control group. However, suppressing TRIM37 expression impeded the effect of PBK overexpression, which could be abrogated by restoration of TRIM37 expression level. Moreover, blocking the NFκB pathway using JSH-23 also counteracted the function of PBK overexpression (Fig. 5a–c). The MTT assay verified that the PBK overexpression-induced olaparib resistance could also be abrogated by TRIM37 knockdown or JSH-23 treatment (Fig. 5d). The effect of the PBK/TRIM37/NFκB axis was further confirmed by the colony formation experiments (Fig. 5e, f). Thus, our results indicated that PBK exerts an olaparib-resistant role through the TRIM37/NFκB axis.

### PBK enhances olaparib resistance through the NFκB pathway in vivo

**Fig. 6:**
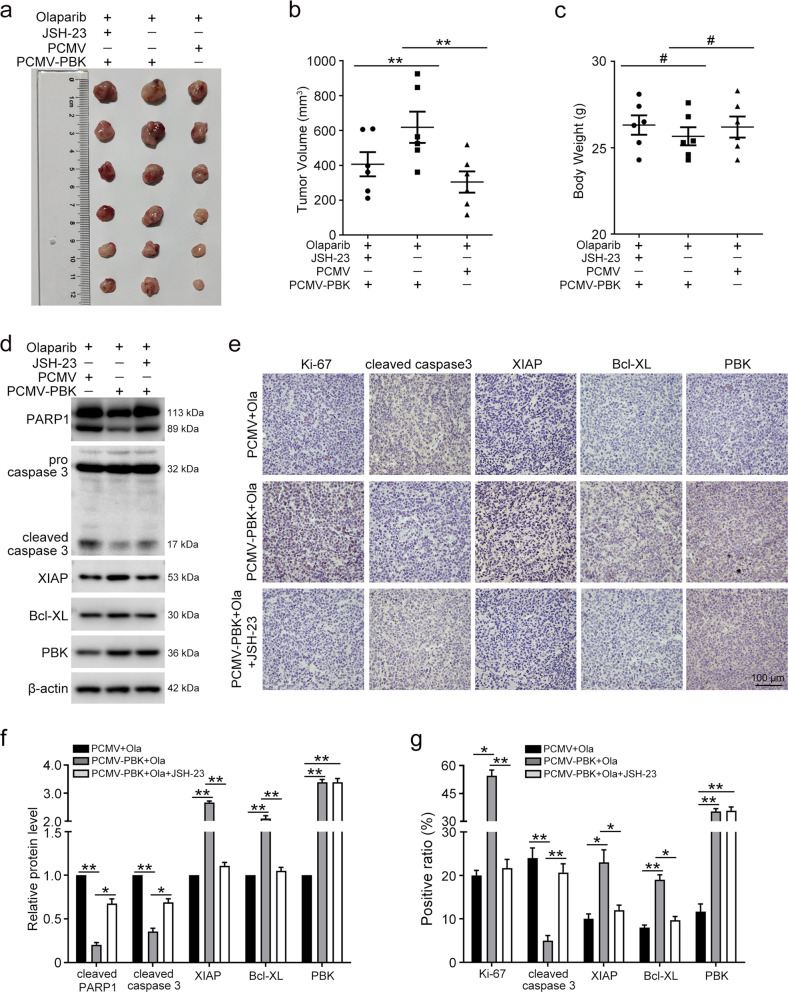
PBK facilitates olaparib resistance in vivo. **a** Photos of subcutaneous tumors. **b** The tumor volumes were computed. **c** The body weights of the mice. **d** Western blot was used to detect the protein levels of PARP1, caspase 3, XIAP, Bcl-XL, PBK, and β-actin in tumor tissues. **e** Representative photographs of IHC staining of Ki-67, cleaved caspase 3, XIAP, Bcl-XL, and PBK in xenograft tumors. **f** Quantification of the protein levels in **d**. **g** Quantification of the positive ratio of Ki-67, cleaved caspase 3, XIAP, Bcl-XL, and PBK in **e**. Scale bar: 100 µm. (Data are presented as the mean ± SEM, ^#^*p* > 0.05, *p < 0.05, **p < 0.01, *n* = 6).

Next, we turned to investigate whether PBK contributes to olaparib resistance in the xenograft mouse model. As shown in Fig. 6a–c, overexpression of PBK enhanced the resistance of UWB1.289 cells to olaparib. However, the administration of JSH-23 blocked the effect of PBK overexpression. The up-regulated apoptotic protein level and NFκB activity evoked by PBK overexpression were also counteracted by using JSH-23 in tumor tissues (Fig. 6d, f). In addition, IHC staining for Ki-67, cleaved caspase3, XIAP, and Bcl-XL validated the reverse function of JSH-23 in the mouse model (Fig. 6e, g). Thus, our data suggested that PBK confers olaparib resistance of ovarian cancer in vivo.

## Discussion

PARPi can induce “synthetic lethality” in homologous recombination deficient (HRD) tumors through inhibiting single-strand DNA repair, resulting in genomic stability and cell death, especially in BRCA1/2 mutation carriers. Interestingly, homologous recombination proficient (HRP) tumors have also been proven to benefit from PARPi^21^. More than 40% of patients harboring BRCA1/2-deficient fail to benefit from PARPi, and most patients will develop resistance to PARPi with prolonged oral administration of PARPi^22^. Many efforts have been made to figure out the mechanism underlying PARPi resistance, such as homologous recombination repair restoration, DNA replication fork protection, reversion mutations of BRCAs, restoration of ADP-ribosylation (PARylation), and loss of 53BP1^23^. However, these explanations still cannot fully reveal the mechanisms of PARPi resistance in ovarian cancer. Here, we reported that the abnormal expression of PBK renders ovarian cancer resistant to olaparib treatment dependent on TRIM37 mediated NFκB activation. PBK was over-expressed in olaparib-resistant ovarian cancer cells and knockdown of PBK increased the apoptotic cell death induced by olaparib. Targeted inhibition of PBK using a specific inhibitor increased the sensitivity of ovarian cancer to olaparib. Our investigation suggested that PBK is an attractive potential target for PARPi resistance diagnosis and chemotherapy.

As a novel serine/threonine protein kinase, many substrates of PBK have been discovered and proven to play important roles in tumor development and progression. PBK was able to phosphorylate ULK1 at Ser469, Ser495, and Ser533 directly and suppress the activity and stability of ULK1, which eventually leads to decreased autophagy and increased temozolomide (TMZ) resistance in glioma^24^. PBK interacts directly with the CHK1 and Cdc25c complex, promotes the phosphorylation of the complex, and further supports the CHK1-mediated maintenance of DNA replication fidelity^25^. In this study, for the first time, we found that TRIM37 is a new substrate of PBK, and the direct interaction between these two proteins promoted the serine phosphorylation of TRIM37. The phosphorylated TRIM37 translocated from the cytoplasm to the nucleus and then activated the NFκB signaling pathway. The PBK/TRIM37/NFκB axis served as a crucial signaling pathway involved in the PARPi resistance of ovarian cancer.

In addition to participating in inflammation and immune response, the ubiquitous NFκB pathway has also been shown to play key roles in cell survival and drug resistance of many malignancies. Chemotherapeutics induced DNA damage response (DDR) could activate the NFκB pathway which protects cells against apoptotic death^26,27^. NFκB contributes to temozolomide (TMZ) resistance in glioblastoma through transcriptionally activating E2F6 expression^28^. Inhibition of NFκB activity suppresses MDR1 mRNA and P-glycoprotein (P-gp) expression, resulting in increasing the sensitivity of resistant colon cancer cells to daunomycin^29^. NFκB pathway contributes to acid ceramidase (AC)-mediated up-regulation of P-gp expression and decreases sensitivity to chemotherapeutic drugs in acute myeloid leukemia (AML)^30^. Here, we reported that olaparib treatment promotes the activation of the NFκB pathway, as indicated by the increased levels of XIAP, Bcl-XL, and p-IκBα. We further found that NFκB activated by PBK/TRIM37 confers resistance to PARPi in ovarian cancer. Inhibition of NFκB activity using JSH-23 counteracted PBK induced chemoresistance both in vitro and in vivo. Thus, the combination of JSH-23 with PARPi might possess a bright future for ovarian cancer treatment.

Up to now, different combination therapies have been invented to enhance the effect of PARPi and overcome PARPi resistance. c-Met, a receptor tyrosine kinase, has been involved in PARPi resistance by restoring HR in a BRCA-independent manner to impair PARPi function. Thus, PARPi in combination with c-Met inhibitors may benefit patients with advanced ovarian cancer^31,32^. The combination of cyclin-dependent kinases (CDK) inhibitors with PARPi is thought to sensitize BRCA-proficient cells to PARPi preclinically^33,34^. A recent phase 1 clinical trial reports that the combination of PI3K inhibitors with PARPi achieves promising results and warrants further clinical evaluation^35,36^. In the current study, PARPi in combination with OTS514 achieved conspicuous results in improving the outcome of PARPi. OTS514 also significantly enhanced the cell apoptosis induced by PARPi treatment. As such, the application of PBK inhibitor provided a potential strategy for sensitizing ovarian cancer to PARPi.

In conclusion, in this study, we demonstrated that PBK directly interacts with TRIM37 to promote its phosphorylation and nuclear translocation, which subsequently activates the NFκB pathway in ovarian cancer cells. In addition, PBK contributed to PARPi resistance of ovarian cancer through the TRIM37/NFκB axis in vitro and in vivo. Our research expanded the explanation of PARPi resistance and provided an experimental basis for overcoming PARPi resistance clinically.

## Supplementary information

Supplementary materials
