# Supplementary material for: PBK drives PARP inhibitor resistance through the TRIM37/NFκB axis in ovarian cancer
Source: Exp Mol Med. 2022 Jul 20;54(7):999–1010. doi: 10.1038/s12276-022-00809-w (PMC9355941; doi:10.1038/s12276-022-00809-w)
Supplement: Supplementary file 1 — Supplementary materials [file 12276_2022_809_MOESM1_ESM.pdf]

Supplementary Figures and Figure legends:

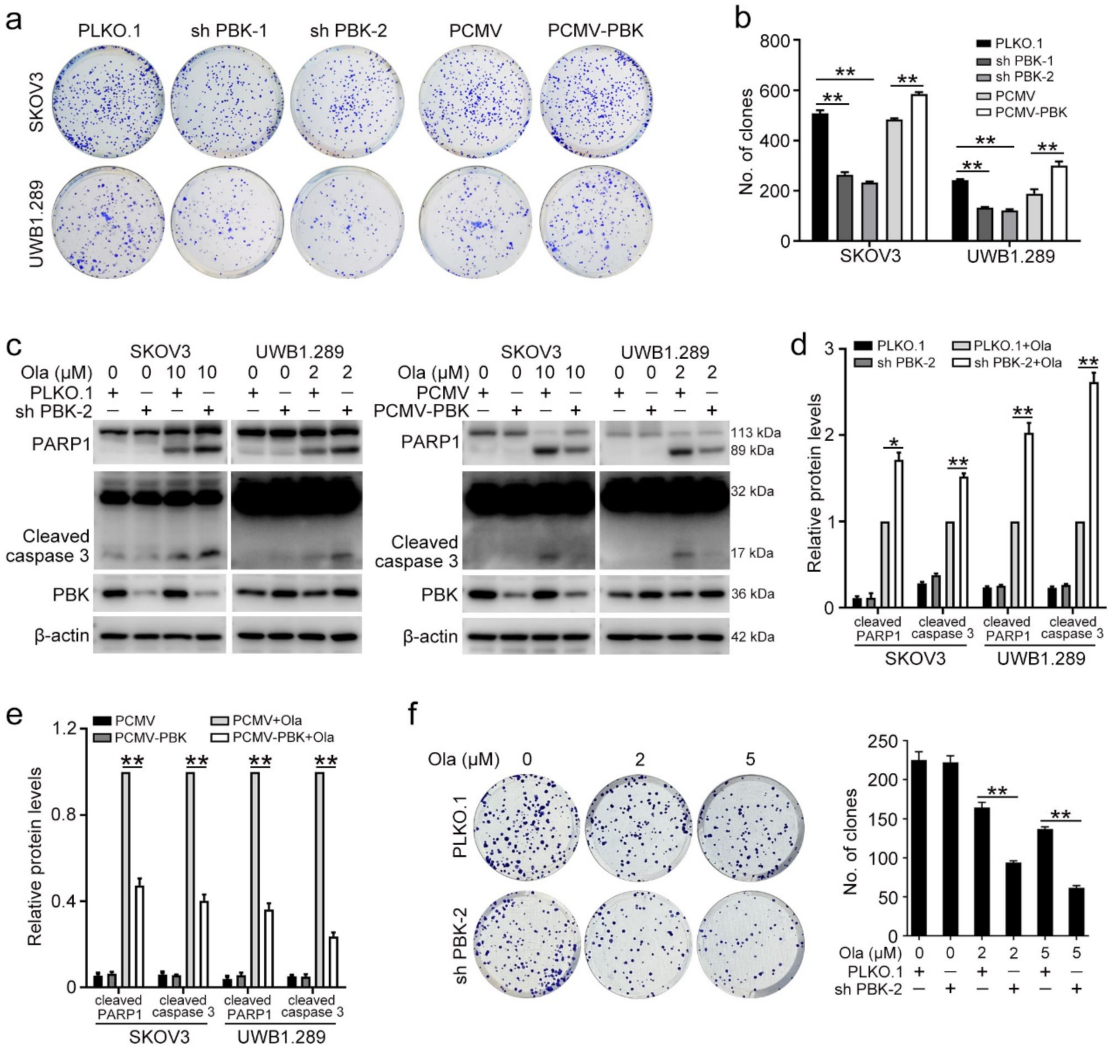

Supplementary Fig. 1. PLKO.1, PBK shRNA 1 (sh PBK-1), PBK shRNA 2 (sh PBK-2), PCMV, and PCMV-PBK plasmids were stably transfected into SKOV3 and UWB1.289 cells. (a) Colony formation assay was conducted to assess the colony formation efficiency of cells in the presence of olaparib (SKOV3, 5 µM; UWB1.289, 1 µM). (b) Quantification of the number of clones in (a). (c) Western blot was conducted to detect the protein levels of PARP1, caspase 3, PBK, and β-actin in cells treated with olaparib for 72 h. (d) and (e) Quantification of the protein levels of cleaved PARP1 and cleaved caspase 3 in (c). (f) SKOV3/Ola cells transfected with PLKO.1 or PBK shRNA 2 (sh PBK-2) were treated with 0, 2, or 5 µM olaparib. Clonogenic assay was used to assess colony formation efficiency. (Data are presented as the mean ± SEM, *$p < 0.05$, **$p < 0.01$, n = 3).

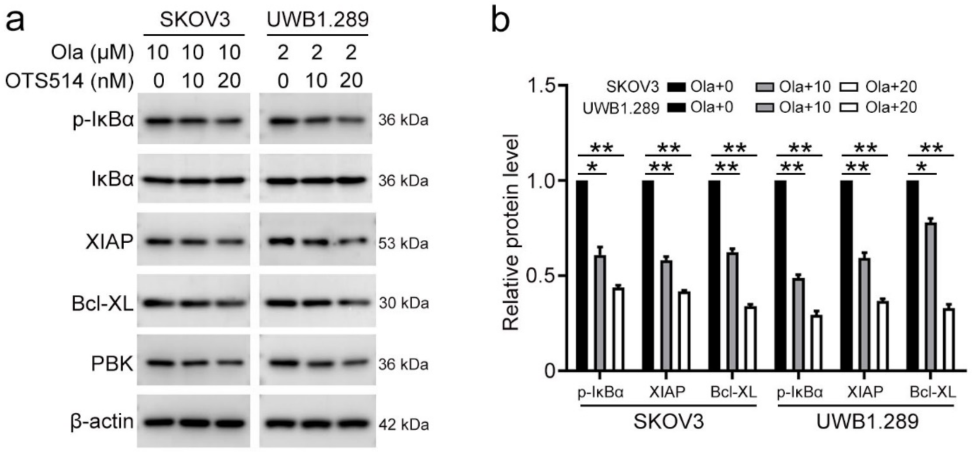

Supplementary Fig. 2. (a) SKOV3 and UWB1.289 cells were challenged with olaparib and/or OTS514 for 24 h, the protein levels of p-IκBα, IκBα, XIAP, Bcl-XL, PBK, and β-actin were detected using western blot. (b) Quantification of the protein levels in (a). (Data are presented as the mean ± SEM, **p* < 0.05, ***p* < 0.01, n = 3).

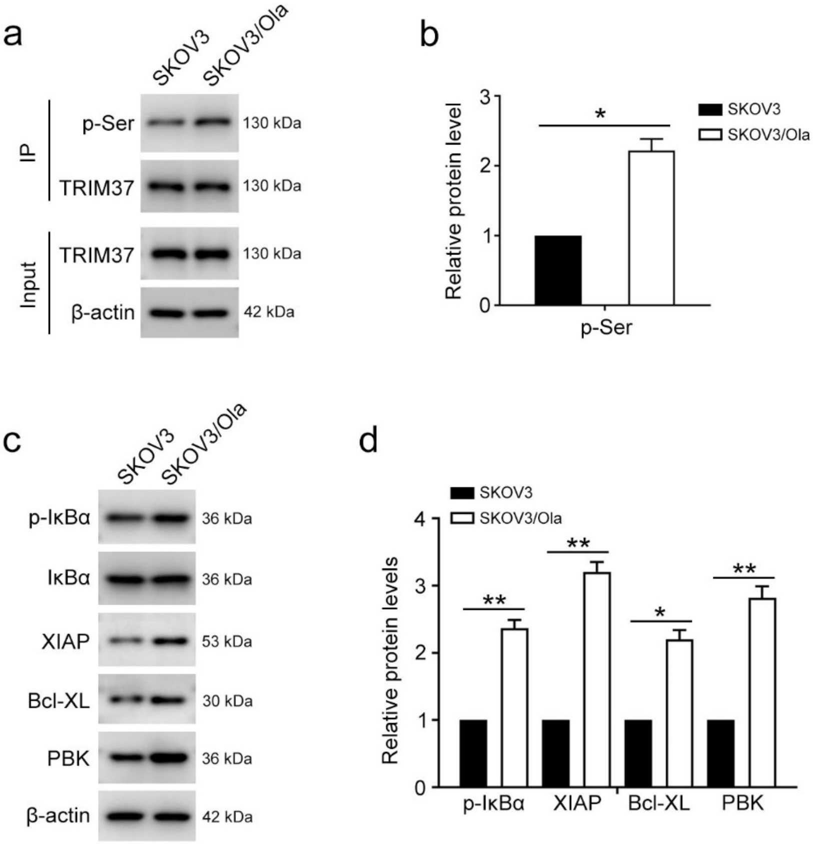

Supplementary Fig. 3. (a) SKOV3 and SKOV3/Ola cells were IPed with anti-TRIM37 antibody and detected with p-serine (p-Ser) and TRIM37 antibodies. (b) Quantification

of the p-Ser level of TRIM37 in (a). (c) Western blot was performed to detect the protein levels of p-IκBα, IκBα, XIAP, Bcl-XL, PBK, and β-actin in SKOV3 and SKOV3/Ola cells. (d) Quantification of the protein levels in (c). (Data are presented as the mean ± SEM, *$p < 0.05$, **$p < 0.01$, n = 3).

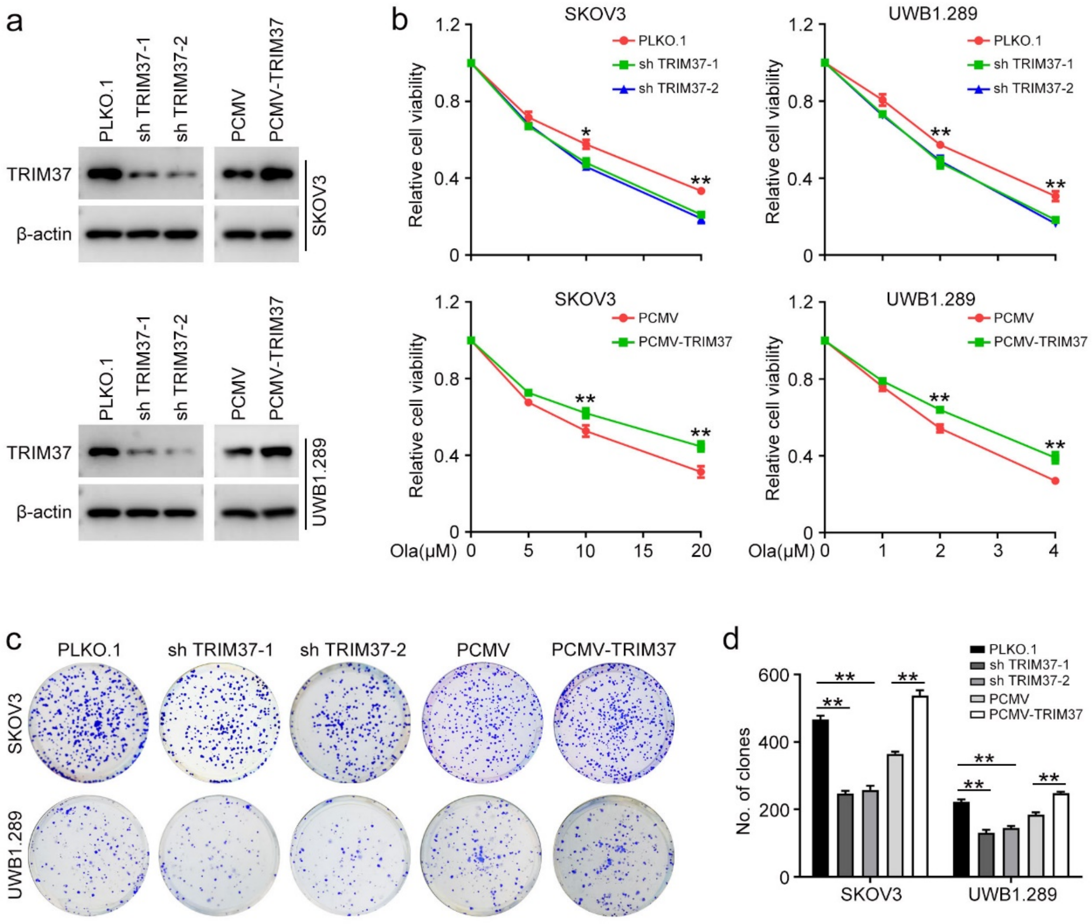

Supplementary Fig. 4. PLKO.1, TRIM37 shRNA 1 (sh TRIM37-1), TRIM37 shRNA 2 (sh TRIM37-2), PCMV, and PCMV-TRIM37 plasmids were stably transfected into SKOV3 and UWB1.289 cells. (a) Western blot was performed to determine TRIM37 protein levels in cells with TRIM37 overexpression or knockdown. (b) The MTT assay was used to detect cell viability in cells treated with olaparib (Ola) for 72 h. (c) Colony formation assay was conducted to assess the colony formation efficiency of cells in the presence of olaparib (SKOV3, 5 μM; UWB1.289, 1 μM). (d) Quantification of the number of clones in (c). (Data are presented as the mean ± SEM, *$p < 0.05$, **$p < 0.01$, n = 3).
